# A novel membrane targeting domain mediates the endosomal or Golgi localization specificity of small GTPases Rab22 and Rab31

**DOI:** 10.1016/j.jbc.2022.102281

**Published:** 2022-07-19

**Authors:** Marcellus J. Banworth, Zhimin Liang, Guangpu Li

**Affiliations:** Department of Biochemistry and Molecular Biology, Peggy and Charles Stephenson Cancer Center, University of Oklahoma Health Sciences Center, Oklahoma City, Oklahoma, USA

**Keywords:** Rab5, Rab22, Rab31, GTPase, endosome, FBS, fetal calf serum, GAP, GTPase-activating protein, GEF, guanine nucleotide exchange factor, gRNA, guide RNA, GST, glutathione S-transferase, HVD, hypervariable domain, ISL, interswitch loop, knockout, KO, MEM, minimal essential medium, MTD, membrane targeting domain, PBS, phosphate-buffered saline, PCR, polymerase chain reaction, RBD, Rab-binding domain, TBS-T, Tris-buffered saline containing 1% Tween 20

## Abstract

Rab22 and Rab31 belong to the Rab5 subfamily of GTPases that regulates endocytic traffic and endosomal sorting. Rab22 and Rab31 (a.k.a. Rab22b) are closely related and share 87% amino acid sequence similarity, but they show distinct intracellular localization and function in the cell. Rab22 is localized to early endosomes and regulates early endosomal recycling, while Rab31 is mostly localized to the Golgi complex with only a small fraction in the endosomes at steady state. The specific determinants that affect this differential localization, however, are unclear. In this study, we identify a novel membrane targeting domain (MTD) consisting of the C-terminal hypervariable domain (HVD), interswitch loop (ISL), and N-terminal domain as a major determinant of endosomal localization for Rab22 and Rab31, as well as Rab5. Rab22 and Rab31 share the same N-terminal domain, but we find Rab22 chimeras with Rab31 HVD exhibit phenotypic Rab31 localization to the Golgi complex, while Rab31 chimeras with the Rab22 HVD localize to early endosomes, similar to wildtype Rab22. We also find that the Rab22 HVD favors interaction with the early endosomal effector protein Rabenosyn-5, which may stabilize the Rab localization to the endosomes. The importance of effector interaction in endosomal localization is further demonstrated by the disruption of Rab22 endosomal localization in Rabenosyn-5 knockout cells and by the shift of Rab31 to the endosomes in Rabenosyn-5-overexpressing cells. Taken together, we have identified a novel MTD that mediates localization of Rab5 subfamily members to early endosomes *via* interaction with an effector such as Rabenosyn-5.

Rab GTPases are important regulators of intracellular membrane trafficking along the endocytic and exocytic pathways (1, 2, 3, 4, 5, 6). Like other Ras-related small GTPases, Rabs alternate between active GTP-bound and inactive GDP-bound conformations, and this canonical GTPase cycle is regulated by GTPase-activating proteins (GAPs) and guanine nucleotide exchange factors (GEFs), which promote GTP hydrolysis and GDP dissociation, respectively (7, 8). Rab GTPases are ancient proteins that exist in all eukaryotes ranging from the last eukaryotic common ancestor to humans (9, 10). In human cells, there are 66 Rabs that localize to different organelles and control multiple vesicular transport steps including vesicle formation, movement and fusion *via* temporal and spatial interactions with specific effectors. However, the mechanism of Rab membrane localization is controversial and remains to be established. All Rabs contain the lipid modification by geranylgeranyl group on C-terminal cysteine residues, which provides necessary hydrophobicity for Rab membrane association but cannot account for membrane localization specificity. Early studies have suggested the C-terminal hypervariable domain (HVD) as the determinant of membrane localization specificity for Rab5 and Rab7 (11), but later studies suggest more complexity involving regions in addition to HVD depending on individual Rabs (12, 13, 14, 15). The steady-state localization of Rabs may require interactions with GEFs, GAPs, and effectors (12, 16).

Membrane localization is essential for Rab function. To further clarify the Rab membrane targeting mechanism, we simplify the question initially by comparing closely related Rabs that share most effectors and regulators but exhibit different membrane localization specificity. To this end, we find Rab22 and Rab31, which are considered isoforms with 87% amino acid sequence similarity and contain essentially the same switch I and II regions for interactions with effectors and regulators (GEFs and GAPs). However, Rab22 is localized to early endosomes, while Rab31 is predominantly in the Golgi complex (17, 18, 19, 20), indicating distinct membrane localization signals. Rab22 and Rab31 belong to the Rab5 subfamily whose members are localized to early endosomes and regulate endosomal sorting, and they share a number of effectors with various binding affinities. Among them are two endosome-tethering factors EEA1 and Rabenosyn-5, which contain both FYVE domain and Rab-binding domain (RBD) to bind PI3P on the early endosomal membrane and interact with the GTP-bound Rab (21, 22). Their independent association with early endosomes *via* PI3P-binding makes them good candidates for establishing and stabilizing a Rab domain on the membrane.

In this study, we identify a novel Rab surface consisting of the N-terminal domain (sequence upstream of the G1 motif), the interswitch loop (ISL) (sequence between the switch 1 and switch 2 regions), and the C-terminal HVD (sequence downstream of the G5 motif), based on their crystal structures (PDB:1YVD-Rab22 and PDB:2FG5-Rab31) (Fig. 1), and propose that this contiguous surface is the Rab membrane targeting domain (MTD) for Rab22 and Rab31 and possibly other Rabs through interaction with effectors and/or regulators directly or indirectly. Our study tests this model.

**Figure 1:**
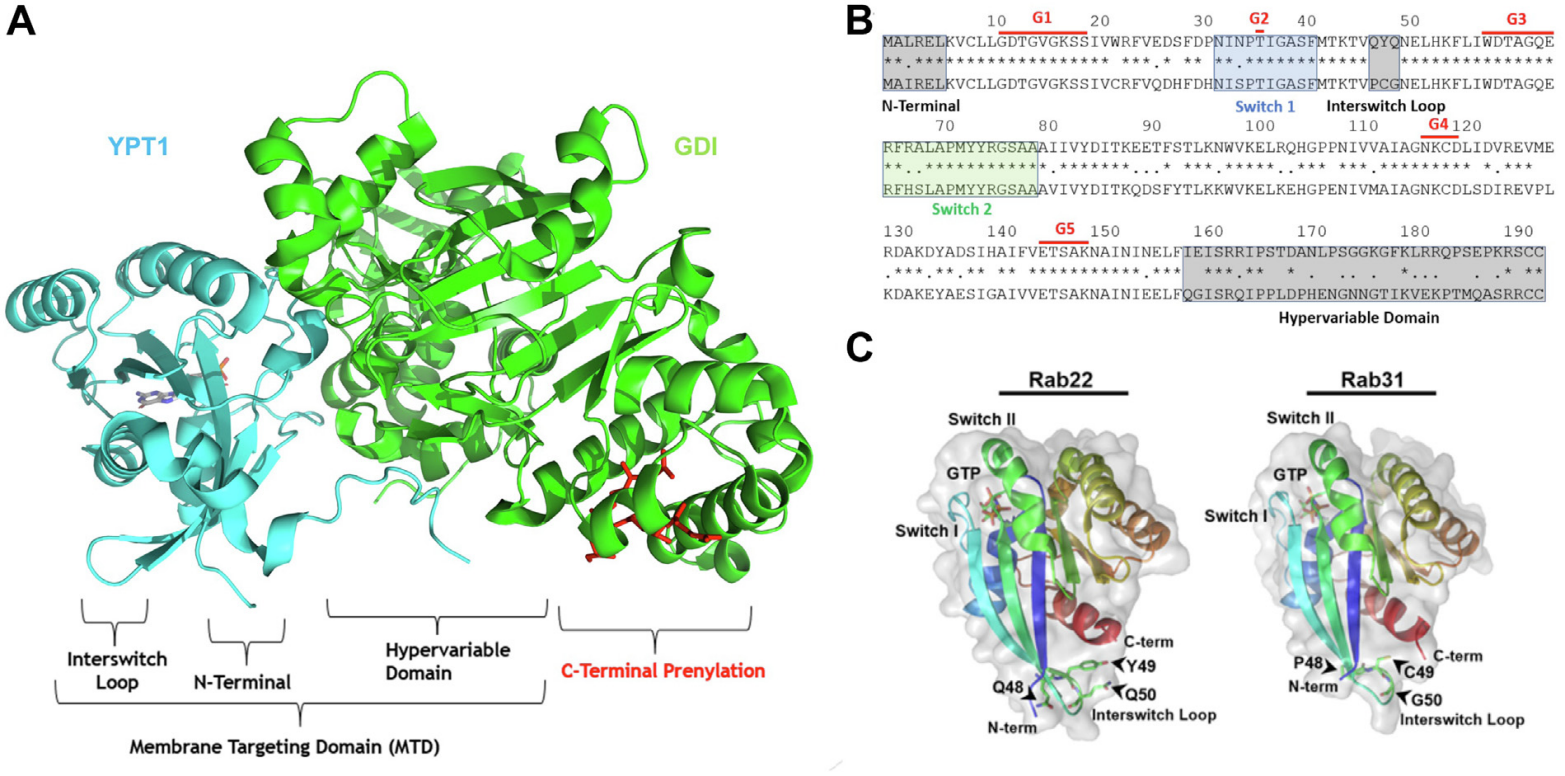
**Mapping of membrane targeting domain (MTD) on YPT1:GDI complex structure and sequence alignment and structural comparison of Rab22 vs. Rab31**. *A*, YPT1:GDI (PDB:2BCG) crystal structure illustrating the structural components of the MTD including the N-terminal domain, interswitch loop (ISL), and C-terminal hypervariable domain (HVD) as indicated. The MTD does not include C-terminal prenylation as it is the same in all Rabs and provides no specificity in membrane targeting. *B*, sequence alignment of Rab22 (*top*) and Rab31 (*bottom*). The *gray boxes* mark the proposed MTD with the N-terminal domain, ISL, and C-terminal HVD, while the *blue* and *green boxes* mark the switch 1 and switch 2 regions for effector interactions, as indicated. G1-G5 indicate the conserved GTP/GDP-binding motifs. *C*, 3D crystal structures of Rab22 (PDB:1YVD) and Rab31 (PDB:2FG5) paired with spatial identification of the various structural regions of the Rabs. The individual components of the MTD are labeled, and their spatial proximity to one another is illustrated. Note that the C-terminal HVD is truncated in these Rab structures.

## Results

### Amino acid sequence differences between Rab22 and Rab31 map to a contiguous structural surface consisting of C-terminal HVD, ISL, and N-terminus

The crystal structure of Ypt1 bound to GDI-1 (RCSB:2BCG) is the most complete Rab structure currently available (23) (Fig. 1*A*), compared to the N-terminus and/or C-terminus truncated Rab structures for crystal stability. Ypt1 is a yeast homolog for human Rab1 and is responsible for proper trafficking of ER-derived vesicles to the Golgi (24, 25). Detailed analysis of the Ypt1-GDI structure revealed three regions in close proximity to one another forming a contiguous 3D surface, which include the N-terminal domain, the ISL, and the C-terminal HVD, and together they may form a potential MTD (Fig. 1*A*). In support of this contention, amino acid sequence alignment of human Rab22 and Rab31 indicated key differences in regions specific to the proposed MTD (Fig. 1*B*). These differences are localized to the C-terminal HVD as well as three residues in the ISL between the switch I and switch II regions, while their N-terminal domains are identical (Fig. 1*B*). Mapping of the proposed MTD motifs onto the crystal structures of Rab22 and Rab31 indicated a similar contiguous 3D surface consisting of the HVD, the ISL, and the N-terminal domain (Fig. 1*C*).

### C-terminal HVD is responsible for distinct Rab22 and Rab31 localization

**Figure 2:**
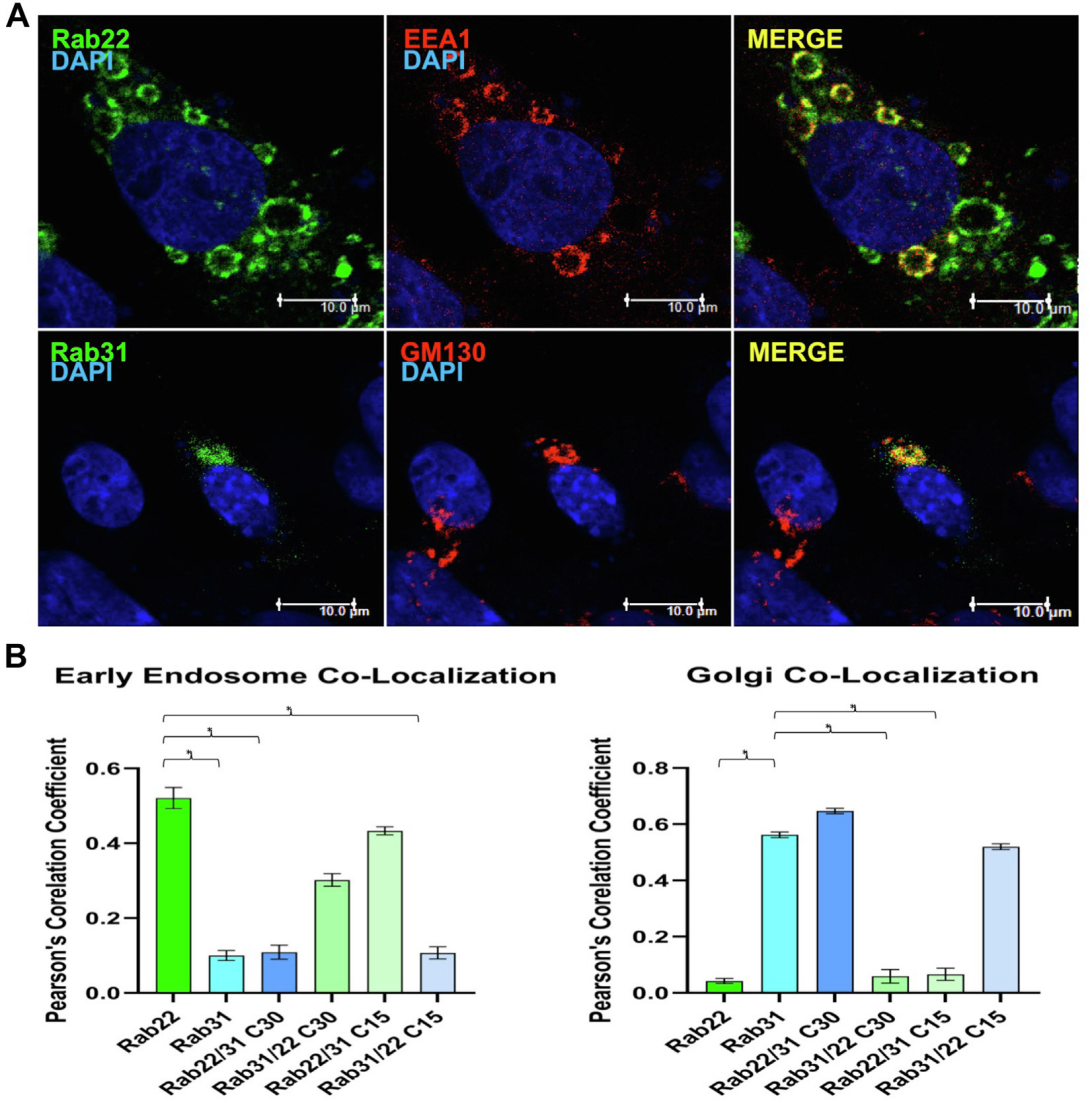
**Distinct intracellular localization of Rab22 and Rab31 in the cell.***A*, shown are confocal immunofluorescence microscopy images of Rab22 (*green*) and Rab31 (*green*) in BHK cells. Rab22 co-localizes with the early endosomal marker EEA1 (*red*), while Rab31 co-localizes with the Golgi marker GM-130 (*red*) as indicated. Of 100 cells examined in each case, more than 80% of cells exhibit the typical phenotype in three independent experiments. Nuclei are indicated by DAPI staining (*blue*). *B*, quantification of co-localization by Pearson’s correlation coefficient. The Pearson’s correlation coefficients are calculated using the confocal images with the built-in co-localization tool in the Improvision Volocity software. Error bars indicate S.E. calculated from data on 10 cells each in three separate experiments, and *t* test analysis is shown with ∗ = *p* < 0.05. The results were reproducible in three independent experiments.

Rabs are conserved across species. We expressed human Rab22 and Rab31 in HEK293 (human), PC12 (rat), and BHK (hamster) cells and determined their intracellular localization by confocal immunofluorescence microscopy. The results consistently showed Rab22 localization on early endosomes marked by EEA1 and Rab31 localization to the Golgi complex marked by GM130. Figure 2 shows typical Rab22 and Rab31 localization patterns in BHK cells. The Rab22 endosomes were enlarged due to enhanced endosome fusion, while Rab31 was mostly found in the perinuclear Golgi structure (Fig. 2), consistent with previous studies (17, 20, 26, 27, 28, 29).

**Figure 3:**
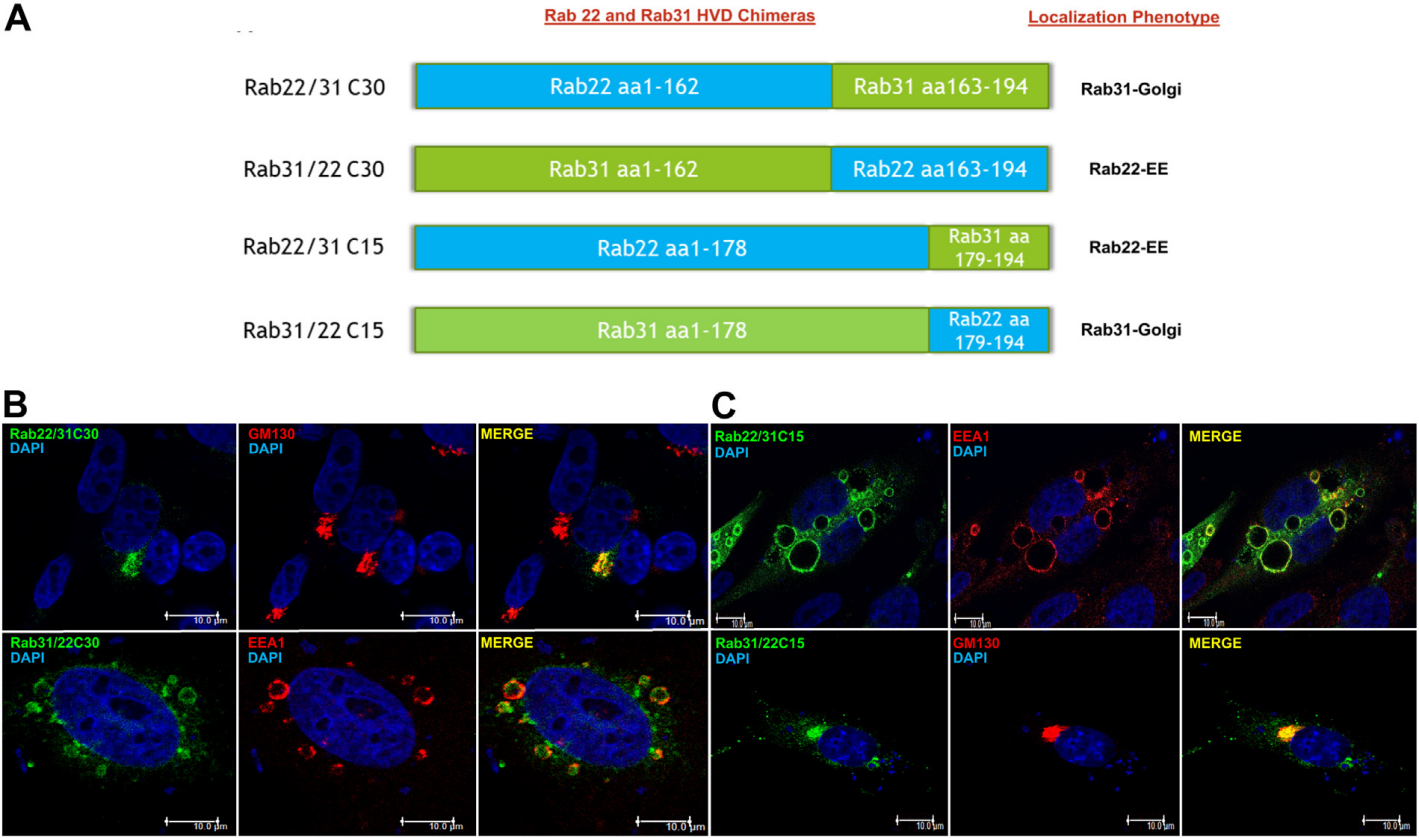
**C-terminal HVD determines localization of Rab22 and Rab31.***A*, schematic illustration of Rab22 (*blue*) and Rab31 (*green*) chimeras. C30 and C15 represent the number of C-terminal HVD residues replaced in the chimeras as indicated. Localization phenotypes of the chimeras are also indicated, in terms of association with early endosomes (EEs) or Golgi and similarity to the corresponding wildtype Rab22 or Rab31. *B*, confocal immunofluorescence microscopy images of Rab22/31C30 and Rab31/22C30 chimeras in BHK cells. Rab22/31C30 (*green*) co-localizes with the Golgi marker GM-130 (*red*), which exhibits perinuclear localization, while Rab31/22C30 (*green*) co-localizes with the early endosomal marker EEA1 (*red*), which exhibits prominent vesicle punctates. *C*, confocal immunofluorescence microscopy images of Rab22/31C15 and Rab31/22C15 chimeras in BHK cells. Rab22/31C15 (*green*) co-localizes with the early endosomal marker EEA1 (*red*), which exhibits prominent vesicle punctates, while Rab31/22C15 (*green*) co-localizes with the Golgi marker GM-130 (*red*), which exhibits perinuclear localization. Overlays of green and red staining (Merge) indicate co-localization (*yellow*), and DAPI staining (*blue*) indicates nuclear location. Of 100 cells examined in each case, more than 80% of cells exhibit the typical phenotype in three independent experiments. Co-localization of each Rab construct with EEA1 or GM130 was quantified and confirmed by Pearson’s correlation coefficient as described in Figure 2*B*. HVD, hypervariable domain.

The putative MTD is composed of the C-terminal HVD, the ISL, and the N-terminal domain (Fig. 1). For Rab22 and Rab31, the N-terminal domain is essentially the same, and thus, we initially focused on the C-terminal HVD and generated a series of Rab22 and Rab31 chimeras with reciprocal replacements in HVD (Fig. 3*A*). These chimeric proteins were expressed in BHK cells, and their intracellular localization was determined by confocal immunofluorescence microscopy. Remarkably the C-terminal 30 residues largely determined the localization of Rab22 or Rab31, i.e., the Rab22 chimeras with Rab31 C-terminal 30 residues (Rab22/31C30) exhibited Rab31 phenotype and co-localized with GM130 in the Golgi, while the Rab31 chimeras with Rab22 C-terminal 30 residues (Rab31/22C30) showed Rab22 phenotype and co-localized with EEA1 in early endosomes (Figs. 3*B* and 2*B*). Indeed, the differential localization of Rab22 and Rab31 and the chimeras was further corroborated by differentiation centrifugation, which showed that Rab22 and the endosome-localized Rab31/22C30 chimera were essentially all in the membrane pellet after 100,000*g* centrifugation, while Rab31 and the Golgi-localized Rab22/31C30 exhibited the same membrane association profile with about 50% in the membrane pellet and 50% in the cytosol fraction (Fig. S1).

To further narrow down the minimal domain responsible for the distinct Rab22 and Rab31 localization, we made and characterized additional chimeras with smaller C-terminal sequence replacements of 15 residues (Rab22/31C15 and Rab31/22C15) and found their localization unchanged and remained the same as Rab22 in the early endosomes and Rab31 in the Golgi complex, respectively (Figs. 3*C* and 2*B*).

**Figure 4:**
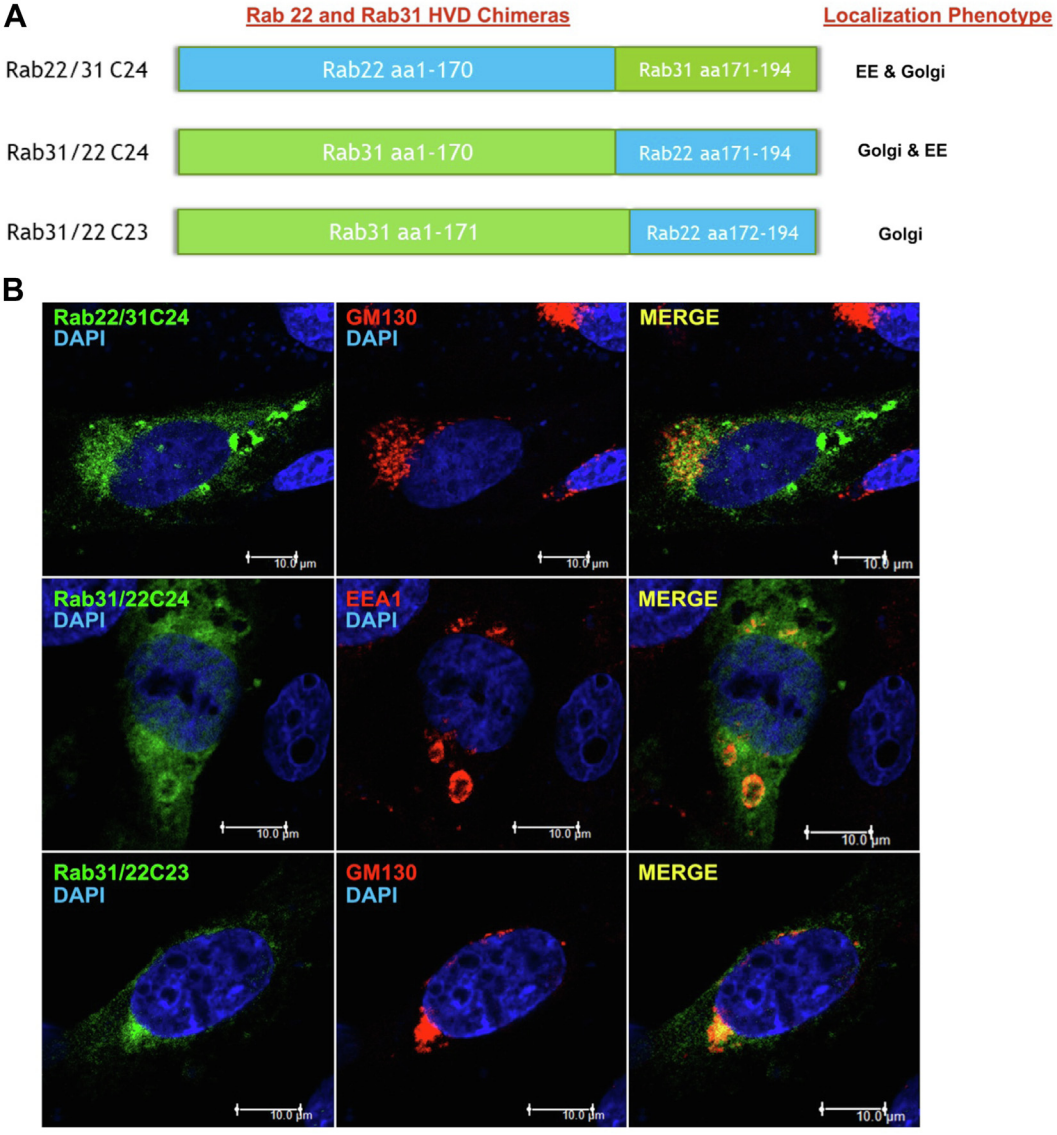
**C-terminal HVD chimeras identify a single residue that affects Rab22 and Rab31 localization.***A*, schematic illustration of Rab22 (*blue*) and Rab31 (*green*) chimeras. C24 and C23 represent the number of C-terminal HVD residues replaced in the chimeras as indicated. Localization phenotype of the chimeras is also indicated on the *right side*, in terms of association with early endosomes (EEs) or Golgi or both. *B*, confocal immunofluorescence microscopy images of Rab22/31C24, Rab31/22C24, and Rab31/22C23 in BHK cells. Rab22/31C24 and Rab31/22C24 (*green*) show partial localization to both EEs and the Golgi, with Rab22/31C24 shifting to the Golgi marked by GM-130 (*red*), while Rab31/22C24 shifting to early endosomes marked by EEA1 (*red*). In contrast, Rab31/22C23 (*green*) co-localizes with the Golgi marker GM-130 (*red*), like Rab31. Overlays of *green* and *red* staining (Merge) indicate co-localization (*yellow*), and DAPI staining (*blue*) indicates nuclear location. Of 100 cells examined in each case, more than 80% of cells exhibit the typical phenotype in three independent experiments. Co-localization of each Rab construct with EEA1 (EE) or GM130 (Golgi) was quantified and confirmed by Pearson’s correlation coefficient with the same approach as described in Figure 2*B*. HVD, hypervariable domain.

These results suggested a sequence between residues 30 and 15 from the C-terminus to be essential for Rab22 and Rab31 localization specificity. Thus, we constructed an additional pair of Rab22 and Rab31 chimeras with reciprocal replacements of C-terminal 24 residues (Rab22/31C24 and Rab31/22C24) (Fig. 4*A*) and determined their intracellular localization by confocal immunofluorescence microscopy. In this case, the C-terminal 24 residues were able to partially shift the localization of Rab22/31C24 toward the Golgi complex marked by GM130 and Rab31/22C24 toward early endosomes marked by EEA1 (Fig. 4*B*). Interestingly, another chimeric construct (Rab31/22C23) (Fig. 4*A*), which had only one amino acid difference from Rab31/22C24, remained as Rab31 localization phenotype in the Golgi (Fig. 4*B*).

**Figure 5:**
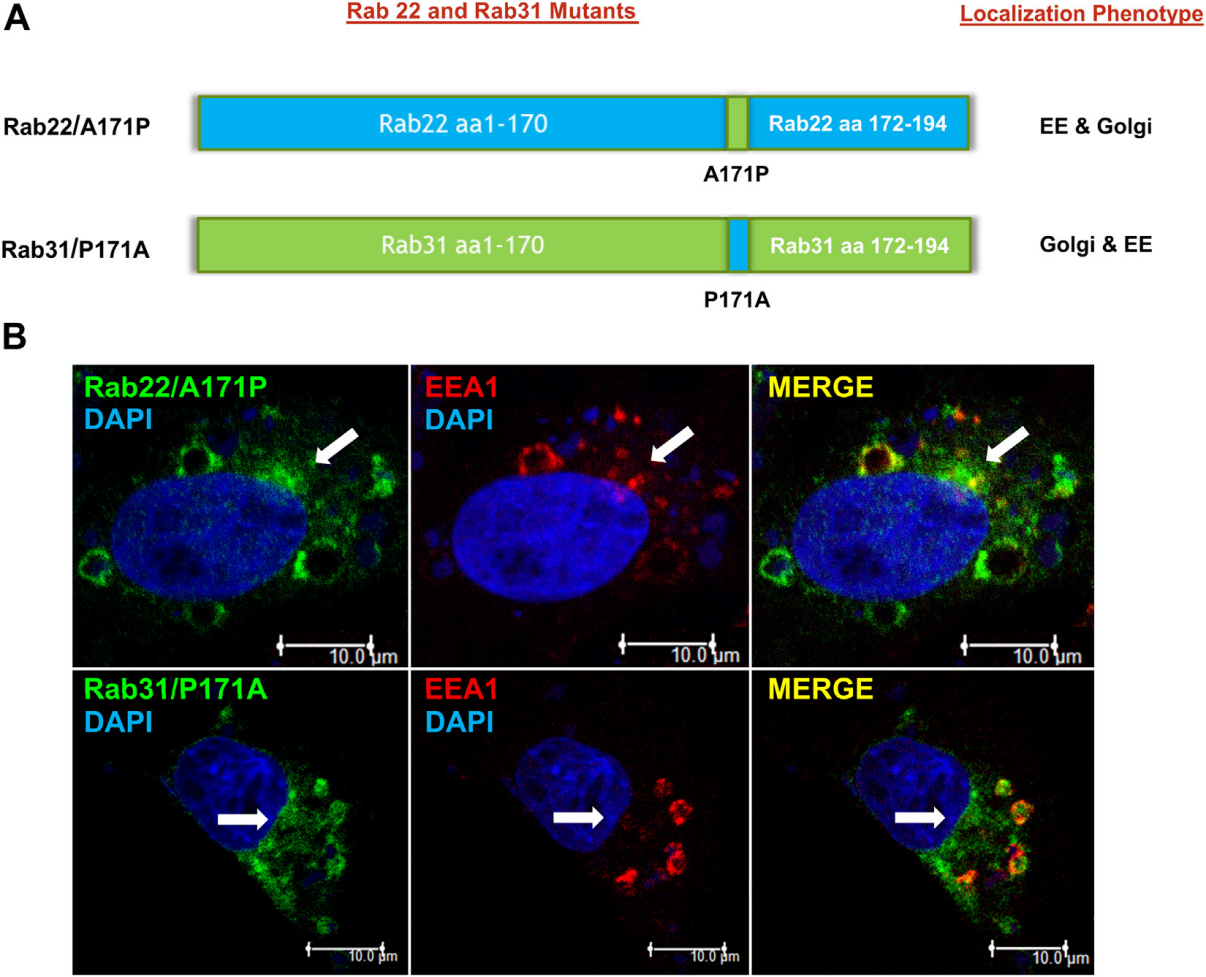
**A single Ala/Pro residue at position 171 of C-terminal HVD can affect the localization of Rab22 and Rab31.***A*, schematic illustration of Rab22 (*blue*) and Rab31 (*green*) mutants at the C-terminal HVD residue 171. Localization phenotype of each mutant is also indicated on the right side, in terms of association with early endosomes (EEs) or Golgi or both. *B*, confocal immunofluorescence microscopy images of Rab22/A171P and Rab31/P171A in BHK cells. Both mutants (*green*) show partial localization to both early endosomes and the Golgi (*arrows*), especially the Rab31/P171A mutant shifting to early endosomes marked by EEA1 (*red*). The Rab22/A171P mutant also shows partial shift to the perinuclear Golgi area (*arrows*), albeit to a lesser extent. Overlays of green and red staining (Merge) indicate co-localization (*yellow*), and DAPI staining (*blue*) indicates nuclear location. Of 100 cells examined in each case, more than 80% of cells exhibit the typical phenotype in three independent experiments. Co-localization of each Rab construct with EEA1 (EE) or GM130 (Golgi) was quantified and confirmed by Pearson’s correlation coefficient with the same approach as described in Figure 2*B*. HVD, hypervariable domain.

These results indicated that a single residue located at position 24 from the C-terminus potentially plays a role in Rab22 and Rab31 localization. This particular residue is Pro in Rab31 and Ala in Rab22 at position 171. We constructed a pair of point mutants at residue 171 (Fig. 5*A*) and tested their localization by confocal immunofluorescence microscopy. Interestingly, the Rab31/P171A mutant, with the replacement of Pro by Ala, showed a partial localization shift to the early endosomes marked by EEA1, while the Rab22/A171P mutant began to show up at the perinuclear Golgi area absent of EEA1 (Fig. 5*B*), suggesting that this HVD residue may directly or indirectly *via* conformation affect interaction with factor(s) essential for membrane localization of Rab22 and Rab31.

### ISL may coordinate with C-terminal HVD within the MTD to facilitate localization and function of Rab22 and Rab31

**Figure 6:**
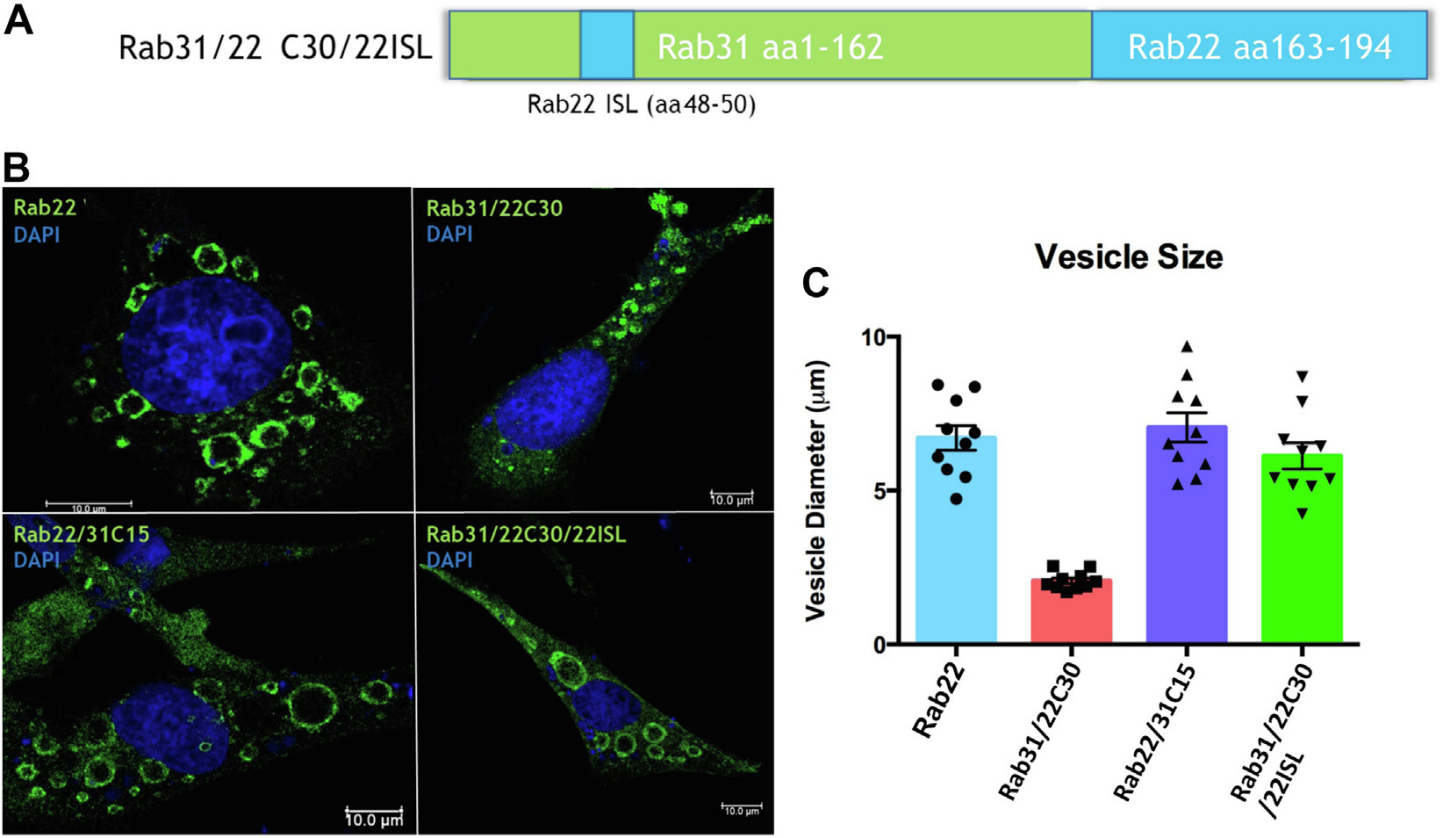
**ISL in the MTD further contributes to Rab endosome association and phenotype.***A*, schematic illustration of the Rab31/22C30/22ISL chimera that contains Rab31 backbone with Rab22 HVD and ISL. *B*, confocal immunofluorescence microscopy images of Rab31/22C30/22ISL in comparison to Rab31/22C30, Rab22/31C15, and Rab22 in BHK cells. Rab31/22C30/22ISL, which contains the complete Rab22 MTD, recapitulates Rab22 localization phenotype with very enlarged endosomes, so is Rab22/31C15. In contrast, Rab31/22C30, which contains only Rab22 HVD without ISL, associates with punctate early endosomes properly but shows less endosomal enlargement. *C*, quantification of the size of largest endosomes in cells expressing each of the indicated Rabs. Error bars indicate S.E. from data on 10 cells of triplicate samples. HVD, hypervariable domain; ISL, interswitch loop; MTD, membrane targeting domain.

We noticed that the chimeric Rab31/22C30 was not only localized to early endosomes but also functional in terms of promoting endosome fusion as evidenced by enlargement of these endosomes. However, the Rab31/22C30 endosomes remained consistently smaller than Rab22 endosomes, suggesting that the Rab31/22C30 interaction with endosomal effectors was not as robust as Rab22. To test this idea and increase Rab31/22C30 functional efficiency, we further transplanted Rab22 ISL onto Rab31/22C30 and found that the resulting chimeric construct Rab31/22C30/22ISL (Fig. 6*A*), which contained both HVD and ISL from Rab22, fully recapitulated Rab22 phenotype in terms of early endosomal localization and morphology (Fig. 6*B*). Indeed, assessment of the vesicle diameter between Rab22, Rab22/31C15, Rab31/22C30, and Rab31/22C30/22ISL endosomes indicated that there was a significant (*p* < 0.0001) increase in the vesicle size of Rab31/22C30/22ISL to the level of Rab22 and Rab22/31C15 compared to Rab31/22C30 (Fig. 6*C*). Along this line, the Rab22/31C15 chimera carried the ISL and functional HVD of Rab22 and thus exhibited the same endosomal morphology as Rab22.

### MTD affects effector interaction with Rab22 and Rab31

**Figure 7:**
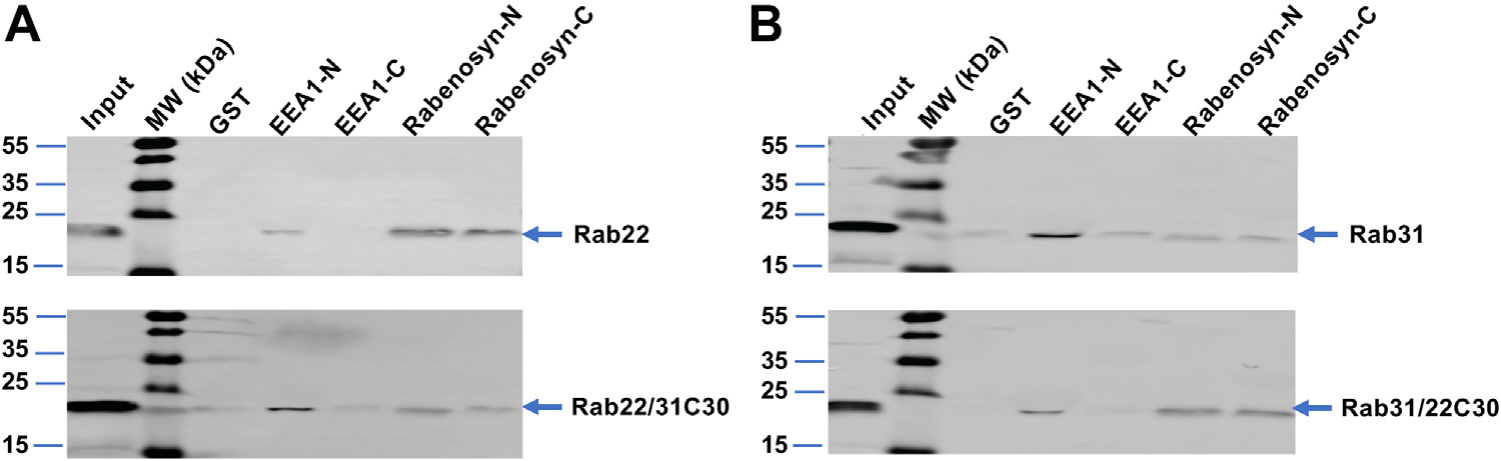
**Differential endosomal effector binding profiles of Rab22 and Rab31 and their chimeras.***A*, effector binding profiles of Rab22 and Rab22/31C30 chimera that contains Rab31 HVD. GST-effector pulldown assays show relative binding strength of EEA1-N, EEA1-C, Rabenosyn-N, and Rabenosyn-C to either Rab, indicating that Rab22 MTD favors stronger binding to Rabenosyn-5 than EEA1. Input indicates the Rab constructs in cell lysates (5% of the amount used for the pulldown). *B*, effector binding profiles of Rab31 and Rab31/22C30 chimera that contains Rab22 HVD. GST-effector pulldown assays show relative binding strength of EEA1-N, EEA1-C, Rabenosyn-N, and Rabenosyn-C to either Rab, indicating Rab31 MTD favors stronger binding to EEA1 than Rabenosyn-5. Input indicates the Rab constructs in cell lysates (5% of the amount used for the pulldown). The bands were quantified by using LI-COR Odyssey Imaging System, and the results were reproducible in two independent experiments. Molecular mass standards (in kDa) are indicated. GST, glutathione S-transferase; HVD, hypervariable domain; MTD, membrane targeting domain.

Among the multiple factors involved in Rab localization, effector interaction was shown to contribute to steady-state Rab localization and required the C-terminal HVD (12). Although Rab5 subfamily members including Rab22 and Rab31 directly contact effectors *via* switch and interswitch regions (30, 31), the C-terminal HVD may also contribute to one or more of these effector interactions directly or indirectly *via* maintenance of proper conformation. To this end, we focused on two early endosome-associated Rab effectors (EEA1 and Rabenosyn-5) that each contains an FYVE domain for binding to PI3P and localization to the early endosome in addition to two RBDs at the N-terminus (EEA1-N or Rabenosyn-N) and the C-terminus (EEA1-C or Rabenosyn-C), respectively (21, 22). We determined if Rab22, Rab31, and the aforementioned chimeras would differentially bind to the RBDs of EEA1 and Rabenosyn-5 by GST pulldown assays, and our data showed that Rab22 and early endosome-localized Rab31/22C30 exhibited stronger binding to Rabenosyn-5, while Rab31 and Golgi-localized Rab22/31C30 favored binding to EEA1-N (Fig. 7). In these experiments, recombinant GST fusion proteins of these EEA1 and Rabenosyn-5 RBDs were affinity-purified on glutathione–Sepharose resin and used to bind Rab22, Rab31 and chimeras expressed in BHK cell lysates, followed by immunoblot analysis of the bound proteins with anti-Rab22 or anti-Rab31 antibodies. One striking observation was that Rab22 showed 2- to 3-fold more robust binding to Rabenosyn-5 than EEA1, while Rab31 exhibited the opposite binding profile for the two effectors (Fig. 7). The C-terminal HVD contributed to the differential binding, as the Rab31 HVD in Rab22/31C30 was able to change its effector binding pattern to that of Rab31 (Fig. 7*A*), while the Rab22 HVD in Rab31/22C30 changed the effector binding pattern to that of Rab22 (Fig. 7*B*). These results were consistent with their intracellular localization suggesting that efficient interaction with Rabenosyn-5 is necessary for the steady-state endosomal localization of Rab22 and the Rab31/22C30 chimera.

### Rabenosyn-5 interaction is necessary for the early endosomal localization of Rab22 and Rab31

**Figure 8:**
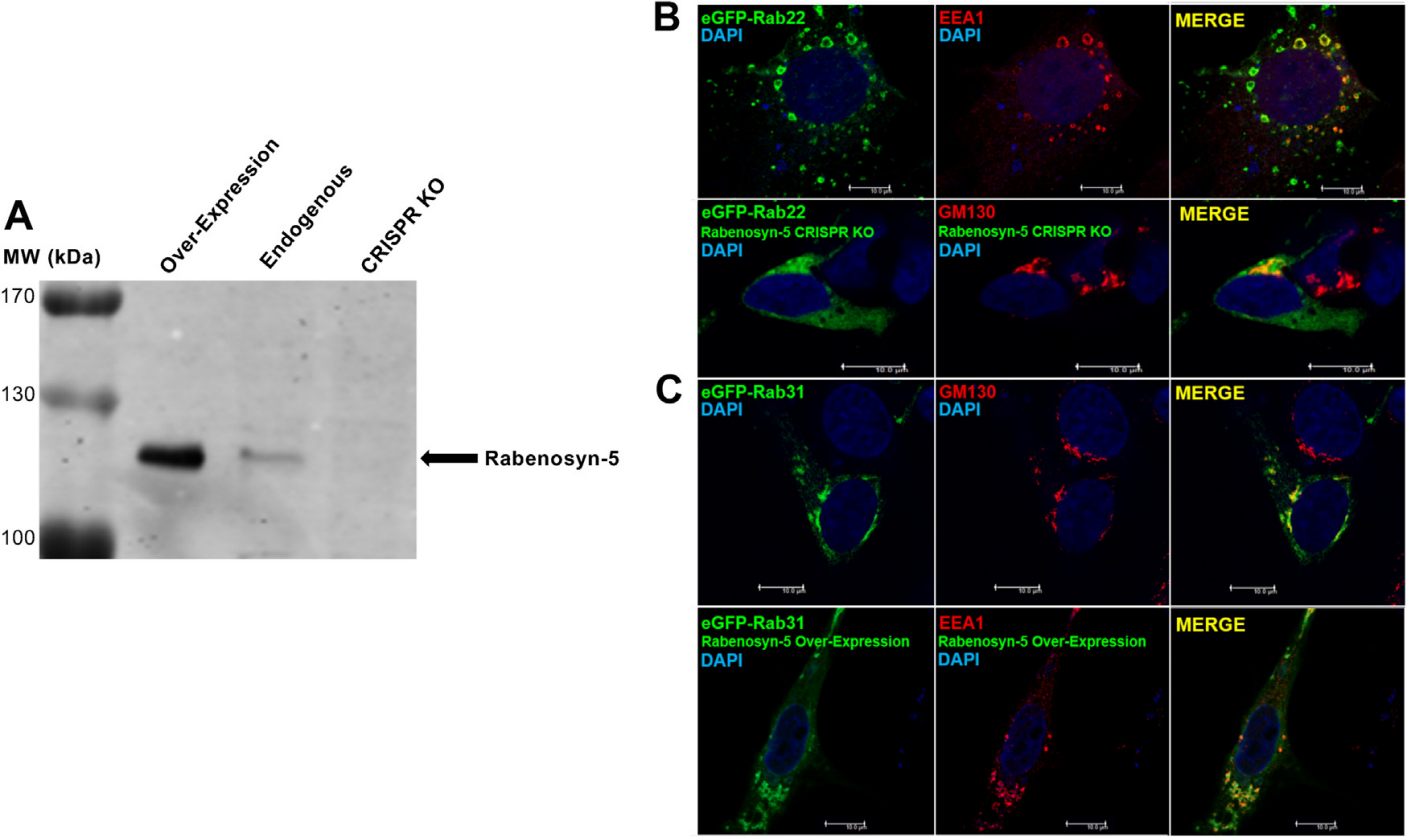
**Rabenosyn-5 necessary for early endosomal localization of the Rabs.***A*, immunoblot analysis of Rabenosyn-5 expression in control (endogenous), overexpression, and CRISPR KO 293 cells. Cell lysates were subjected to SDS-PAGE, and proteins were transferred to immobilon-P membranes and probed with the anti-Rabenosyn-5 antibody. The bands were quantified by using LI-COR Odyssey Imaging System, and the Rabenosyn-5 overexpression was 8-fold over the endogenous level. The results were reproducible in two independent experiments. Molecular mass standards (in kDa) are indicated. *B* and *C*, confocal fluorescence microscopy images of eGFP-Rab22 or eGFP-Rab31 in control, Rabenosyn-5 overexpression, or Rabenosyn-5 KO cells. In control cells, eGFP-Rab22 and eGFP-Rab31 localize to early endosomes marked by EEA1 and the perinuclear Golgi marked by GM-130, respectively. In Rabenosyn-5 KO cells, eGFP-Rab22 diminishes its signature punctate endosomal localization and shifts to perinuclear Golgi area marked by GM-130, while in Rabenosyn-5 overexpression cells, eGFP-Rab31 partially shifts to early endosomes marked by EEA1, as indicated. Of 100 cells examined in each case, more than 80% of cells exhibit the typical phenotype in three independent experiments. Co-localization of each Rab construct with EEA1 or GM130 was quantified and confirmed by Pearson’s correlation coefficient with the same approach as described in Figure 2*B*.

The aforementioned data suggested differences between Rab22 and Rab31 in terms of interaction with early endosomal effectors, and insufficient interaction with Rabenosyn-5 could fail to stabilize Rab31 on early endosomes, which instead was relocated to the Golgi complex as a putative default pathway and destination (13, 15). To test this contention, we overexpressed or knocked out Rabenosyn-5 in HEK293 cells and determined the effects on Rab31 and Rab22 localization by confocal microscopy. The data showed that Rabenosyn-5 overexpression (Fig. 8*A*) was able to partially move Rab31 to early endosomes marked by EEA1 (Fig. 8*B*), while Rabenosyn-5 depletion by CRISPR-Cas9 approach (Fig. 8*A*) disrupted the early endosomal localization of Rab22 and instead shifted its localization to the Golgi complex (Fig. 8*B*). This suggests the Rabenosyn-5 level in the cell may play a key role in the localization of both Rab22 and Rab31, with efficient Rabenosyn-5 interaction necessary for proper endosomal localization.

### Rab5 MTD transplantation to Rab31 shifts its localization to early endosomes

**Figure 9:**
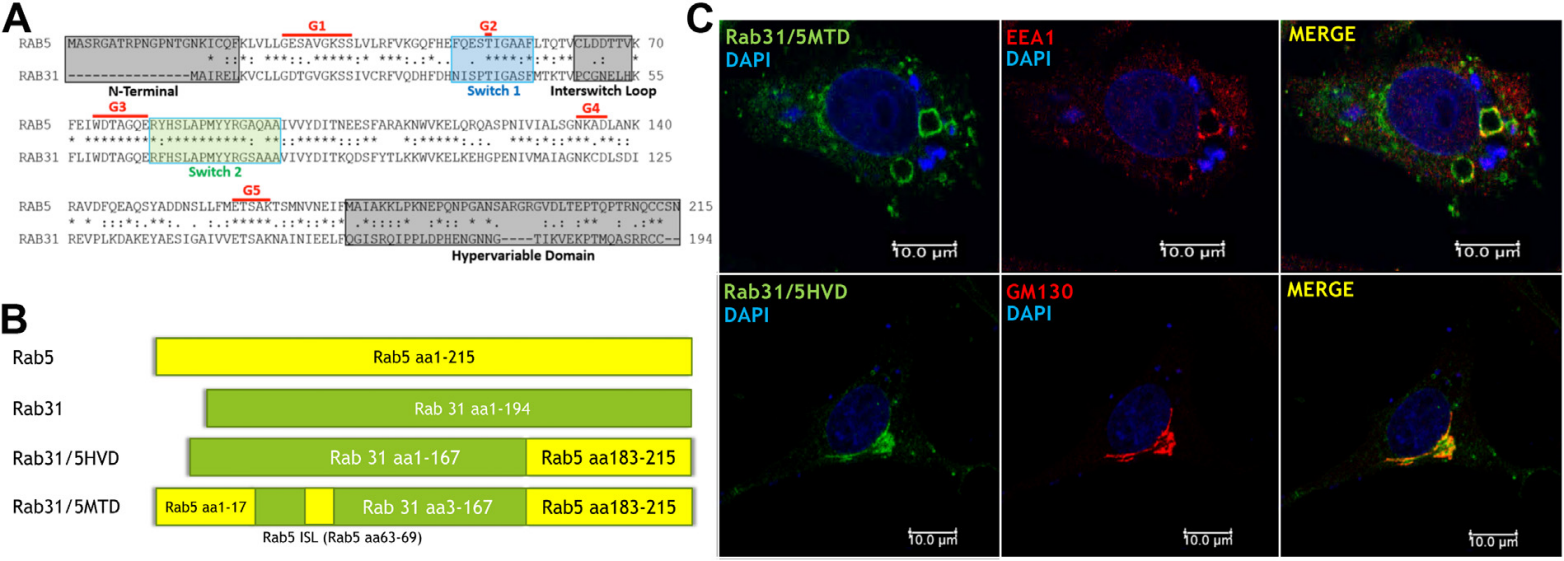
**Sequence alignment of Rab5 and Rab31 and schematic illustration and intracellular localization of their chimeras.***A*, sequence alignment of Rab5 and Rab31. The *gray boxes* mark the proposed MTD including the N-terminal domain, ISL, and C-terminal HVD, while the *blue* and *green boxes* mark the switch 1 and switch 2 regions involved in effector interactions, as indicated. G1-G5 indicate the conserved GTP/GDP-binding motifs. *B*, schematic illustration of Rab5 (*yellow*) and Rab31 (*green*) and their HVD and MTD chimeras as indicated. *C*, confocal immunofluorescence microscopy images of Rab31/5MTD and Rab31/5HVD in BHK cells. Rab31/5MTD (*green*) shifts to enlarged early endosomes containing EEA1 (*red*), while Rab31/5HVD largely remains in the perinuclear Golgi area marked by GM-130 (*red*). Of 100 cells examined in each case, more than 80% cells exhibit the typical phenotype in three independent experiments. Co-localization of each Rab construct with EEA1 or GM130 was quantified and confirmed by Pearson’s correlation coefficient with the same approach as described in Figure 2*B*. HVD, hypervariable domain; ISL, interswitch loop; MTD, membrane targeting domain.

As part of the early endosomal Rab MTD, the C-terminal HVD of Rab22 was shown to confer enhanced binding of Rabenosyn-5 and endosomal localization to Rab31 as described earlier. We further tested whether this MTD concept is applicable to other early endosomal Rabs such as Rab5, which regulates early endosome fusion and entry to the lysosomal degradation pathway (6, 32). To this end, we transplanted the C-terminal HVD or the entire MTD of Rab5a to Rab31 and determined if it would be sufficient for relocalization of Rab31 to the early endosomes. These two Rabs share only 40% amino acid identity. Based on their crystal structures (PDB:1TU4 for Rab5 and PDB:2FG5 for Rab31) and sequence alignment to define the MTD (Fig. 9*A*), we generated Rab31/5HVD and Rab31/5MTD chimeras (Fig. 9*B*), expressed them in BHK cells, and determined their intracellular localization by confocal immunofluorescence microscopy (Fig. 9*C*). Unlike Rab22 HVD, the Rab5 HVD alone was not sufficient in moving Rab31 to early endosomes as the Rab31/5HVD chimera remained at the Golgi complex (Fig. 9*C*). In contrast, the Rab31/5MTD chimera, which contained the complete Rab5 MTD, was able to localize to the early endosomes marked by EEA1 (Fig. 9*C*). Like Rab22, these Rab31/5 MTD endosomes were enlarged, most likely due to the fact that Rab31 exhibits much lower intrinsic GTPase activity compared to Rab5 and is mostly in the active GTP-bound conformation, like Rab22 that shares the same GTPase domain (Fig. S2).

### Rab5 MTD transplantation to Rab31 enhances its interaction with Rabenosyn-5

**Figure 10:**
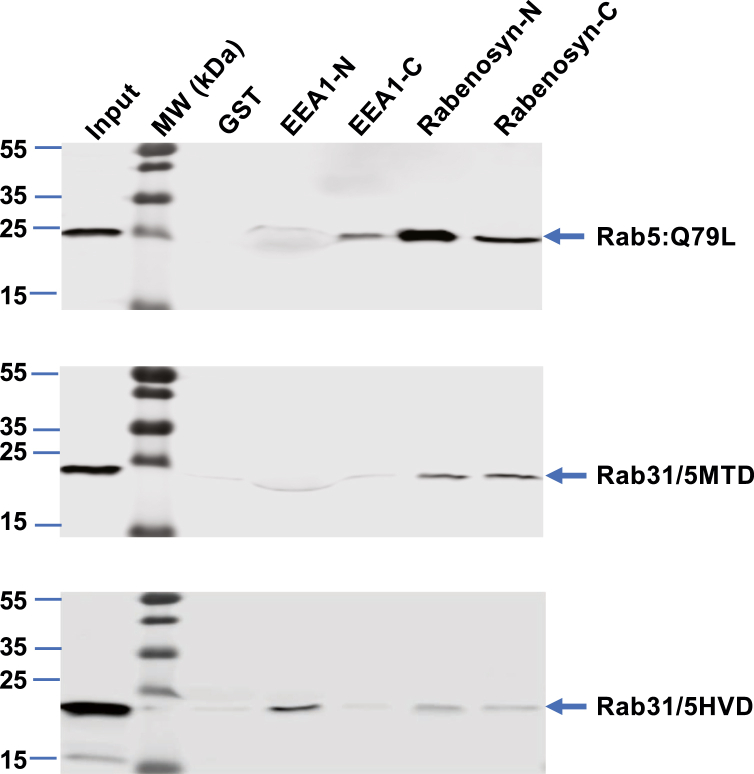
**Differential endosomal effector binding profiles of Rab5 and Rab31 chimeras that contain Rab5 MTD or HVD.** GST-effector pulldown assays show relative binding strength of EEA1-N, EEA1-C, Rabenosyn-N, and Rabenosyn-C to the indicated Rabs, including Rab5:Q79L, Rab31/5MTD, and Rab31/5HVD. The Rabs that contain the endosomal MTD from Rab5 (Rab5:Q79L and Rab31/5MTD) favor stronger binding to Rabenosyn-5, like Rab22, while Rab31/5HVD that lacks complete endosomal MTD shows reduced binding to Rabenosyn-5 but enhanced binding to EEA1, like Rab31. Input indicates the total Rab constructs in cell lysates (5% of the amount used for the pulldown). The bands were quantified by using LI-COR Odyssey Imaging System and the results were reproducible in two independent experiments. Molecular mass standards (in kDa) are indicated. GST, glutathione S-transferase; HVD, hypervariable domain; MTD, membrane targeting domain.

We further demonstrated that replacement of Rab31 MTD with that of Rab5 was able to enhance its interaction with Rabenosyn-5 while reducing EEA1 interaction (Fig. 10), consistent with the Rab31/5MTD localization to the early endosomes (Fig. 9). Using the aforementioned GST-effector fusion proteins, we did pulldown assays with the Rab31/5HVD and Rab31/5MTD chimeras in comparison with Rab5:Q79L, which is a constitutively active, GTP-bound form of Rab5 (33, 34) and served as a positive control for early endosome localization and effector binding profile. Like Rab22, Rab5:Q79L showed stronger binding to Rabenosyn-5 than EEA1 (Fig. 10). In this regard, the endosome-associated Rab31/5MTD but not Rab31/5HVD exhibited the same binding profile as Rab5:Q79L (Fig. 10), in support of our model that enhanced binding affinity to the endosomal effector Rabenosyn-5 may account for Rab31/5MTD localization to the early endosomes. This MTD concept is also consistent with a previous study suggesting an important function of non-HVD sequences in Rab5 interaction with effectors EEA1 and Rabaptin-5 (12).

## Discussion

Rab GTPases are localized to each organelle on the endocytic and exocytic pathways and responsible for proper membrane trafficking in eukaryotic cells. Rab5 subfamily members in particular are early endosomal Rabs and control endocytic trafficking or recycling. Among them, Rab31 appears an outlier in the sense that it is mostly associated with the Golgi at steady state, with only a small fraction on early endosomes which could increase upon EGF signal transduction (35) or phagocytosis (36). On the other hand, its closest relative Rab22 is localized to the early endosome and regulates recycling, although it shares nearly 90% sequence similarity with Rab31. Through sequence alignment and structural comparison of Rab22 and Rab31, we have identified a novel MTD responsible for early endosomal localization of these Rab5 family members. The MTD consists of the N-terminal domain, the ISL, and the C-terminal HVD. For Rab22 and Rab31, the N-terminal domain is the same, while the C-terminal HVD plays a key role in determining their steady-state localization at the early endosome. For Rab5 and Rab31, there are less than 40% sequence similarity and significant differences in all components of MTD. In this case, the transplantation of entire Rab5 MTD to Rab31 is necessary and sufficient to move Rab31 from Golgi to early endosomes. The MTD concept is thus consistent with earlier findings that have suggested HVD as well as other regions for membrane localization of various Rabs (11, 12, 13, 14, 15) and may provide a unified mechanism for Rab membrane localization. A caveat is that we generate the Rab constructs and overexpress them in the cell, which might affect protein localization to some degree. While we cannot completely rule out this possibility, our data on Rab22 and Rab31 localization recapitulate previous reports on their endogenous counterparts in early endosomes and the Golgi complex, respectively (18, 28, 37), suggesting that the overexpression level in our study does not significantly alter the localization of these Rabs. Like Rab5, however, overexpression of the endosome-associated Rab22 can increase the size of early endosomes due to increased membrane fusion, as shown in this study as well as a previous report (26). In this regard, Rab31 shows no such activity, lending further support to its distinct localization and function from Rab22.

**Figure 11:**
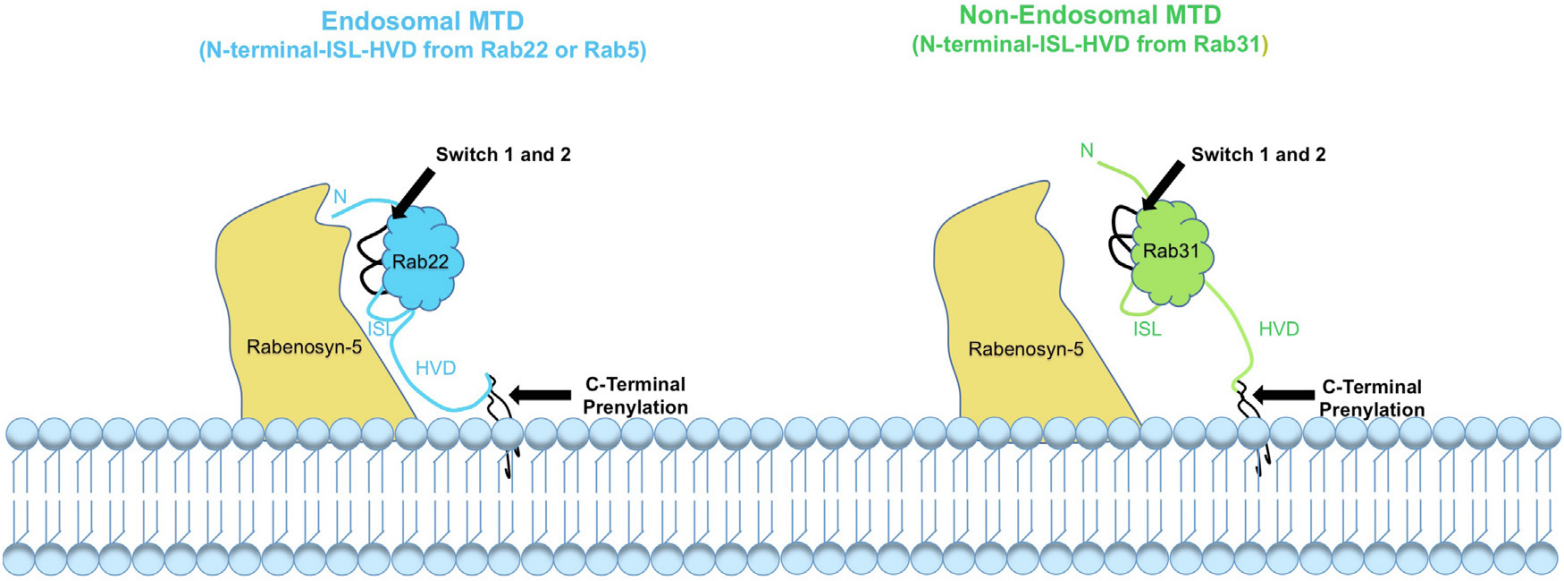
**MTD model for Rab endosomal membrane localization.** The putative early endosomal MTD of Rab22 or Rab5 includes the N-terminal domain, the ISL, and the C-terminal HVD that together form a contiguous structural surface for robust interaction with the early endosomal effector Rabenosyn-5 directly or indirectly *via* conformation of the switch regions, which may contribute to stabilization of the Rabs on early endosomes. Rabenosyn-5 is a resident early endosomal protein *via* binding to the membrane lipid PI3P. The Rab31 MTD, on the other hand, shows reduced interaction with Rabenosyn-5 and thus presents a weaker signal for early endosome localization. This model does not mutually exclude the contribution of other factors such as a GEF or GDF. C-terminal prenylation is essential for membrane association but not part of the MTD as it is present in all Rabs and provides no specificity. GEF, guanine nucleotide exchange factor; HVD, hypervariable domain; ISL, interswitch loop; MTD, membrane targeting domain.

As a component of MTD, the C-terminal HVD is necessary but not sufficient for steady-state endosomal localization of the Rabs, as evidenced by our data on the difference between Rab31/5 HVD and Rab31/5 MTD chimeras. The MTD may contribute directly or indirectly *via* maintaining proper conformation to interact with the factors for membrane localization, including GEFs, GDFs and/or effectors. While interaction with a GEF and/or a GDF may recruit an endosomal Rab to the membrane (16, 38), our data indicate that interaction with Rabenosyn-5 is essential for steady-state localization at the early endosome, consistent with a previous report that effector interaction is necessary for Rab9 localization at the late endosome (12). Indeed, the MTD contributes to interaction with the effectors, and Rab5 and Rab22 MTDs show the same effector binding profiles for early endosomal localization, while the Rab31 MTD disrupts such effector interaction (Fig. 11) and leads to its relocalization to the Golgi complex. While MTD might also contribute to interactions with other factors such as GEFs, our data demonstrate its role in effector interaction and the importance of such interaction in early endosomal localization. In this regard, Rab31 localization appears dynamic and shows increased association with early endosomes upon Rabenosyn-5 overexpression, which may rescue the insufficient effector interaction. Indeed, our data are consistent with previous reports that show Rab31 function on early endosomes upon EGF stimulation (35) and on phagosomes during phagocytosis (36). On the other hand, insufficient Rabenosyn-5 interaction results in Rab31 localization to the Golgi complex, so is the Rab22 localization in Rabenosyn-5 knockout (KO) cells. Thus, the Golgi localization appears a default destination for these early endosomal Rabs when they are not sufficiently stabilized on the early endosome. It is interesting to note that these endosomal Rabs tend to move to and accumulate at the Golgi complex when there is insufficient effector interaction, in support of the contention of a default pathway to the Golgi proposed for other Rabs (13, 15), although the mechanism is not yet clear.

Rabenosyn-5 is a long helical tethering molecule and contains two RBDs at the N- and C-termini, respectively (21, 30). Importantly, it also contains an FYVE motif in the C-terminal domain for binding PI3P and association with early endosomes independent of Rab interaction. Along this line, our data suggest that the early endosomal resident Rabenosyn-5 plays a key role in stabilization of the Rab5 family of GTPases on early endosomes *via* interaction with the proposed MTD (Fig. 11).

## Experimental procedures

### Construction of chimeras and site-directed mutagenesis

To generate Rab22/Rab31 C-terminal chimeras, the wildtype Rab22 backbone was shortened 30, 24, 23, 15 amino acids from its C-terminus and replaced by the C-terminal counterparts of Rab31, respectively, by PCR. For expression in mammalian cells, the resulting chimera constructs, Rab22/31C30, Rab22/31C24, Rab22/31C23, and Rab22/31C15, were cloned into the MluI/NheI sites of the pBI expression vector (Takara Bio). Using the same strategy, we generated a reciprocal set of Rab31/Rab22C chimeras, including pBI/Rab31/22C30, pBI/Rab31/22C24, pBI/Rab31/22C23, and pBI/Rab31/22C15. Similarly, Rab31 and Rab5 chimeric constructs were generated. Furthermore, site-directed mutagenesis was employed to generate pBI/Rab22/31C30/31ISL, pBI/Rab31/22C30/22ISL, pBI/Rab22/A171P, and pBI/Rab31/P171A. These Rab chimeras were also cloned with N-terminal Myc or GFP tag and validated by DNA sequencing. The resulting chimeric proteins were expressed in cell cultures and identified by immunoblot analysis with respective Rab, Myc, and GFP antibodies as detailed below.

### Cell culture and transfection

BHK-21 cell monolayers were grown in 35-cm tissue culture dishes with 2 ml of Eagle’s minimal essential medium (MEM) containing 5% fetal bovine serum (FBS), L-glutamine, and Antibiotic-Antimycotic (Invitrogen) and cultured in humidified 37 °C incubators with 5% CO_2_. For protein expression, the cell monolayers were co-transfected with the indicated plasmid constructs and pTet-off (1:1) *via* the Lipofectamine 2000–mediated procedure (Invitrogen). At 24 h post transfection, cell lysates were directly collected for protein expression detection by immunoblot analysis. For CRISPR-Cas9-mediated Rabenosyn-5 KO, two guide (g) RNAs were expressed, respectively, with Cas9 in HEK293 (human embryonic kidney) cells, including 5′ CACCGTCAGCTTCACTCACATTACG 3′ and 5′ CACCGGGGGATGATCGAGCAGAGTC 3′ targeting exon 4 and exon 5 of Rabenosyn-5, respectively. Their cDNAs were cloned into the Lenti-CRISPR-V2 vector (39) and validated by DNA sequencing. The resulting constructs were transfected into HEK293 cells *via* the Lipofectamine 2000–mediated procedure (Invitrogen), and the cells were selected with 5 μg/ml of puromycin in DMEM containing 5% FBS, L-glutamine, and Antibiotic-Antimycotic (Invitrogen). After 24 h of incubation in humidified 37 °C incubators with 5% CO_2_, individual surviving cells were sorted and isolated into 96-well tissue culture plates and grown into subsequent larger plates until individual lineages were established for sample collection and validation by genomic DNA sequencing and immunoblot analysis.

### Immunoblot analysis

The aforementioned transfected cells or Rabenosyn-5 KO cells were washed once with phosphate-buffered saline (PBS) and lysed in SDS sample buffer (50 Mm Tris-Cl (pH 6.8), 2% SDS, 0.1% bromophenol blue, 10% glycerol) supplemented with fresh 100 mM 2-mercaptoethanol. Lysates were denatured by boiling for 3 min and subjected to 16% SDS-PAGE analysis. Then, proteins were transferred to Immobilon-P membranes (PVDF, Millipore), which were blocked with 8% dry milk in TBS-T (1% Tween20) and then probed with specific primary antibodies, including those for Rabenosyn-5 (Abcam), Rab22 (Abcam), Rab31 (Sigma), Rab5 (Calico Biolabs), Myc (Invitrogen), and GFP (Invitrogen). The membrane was then washed three times in TBS-T (5 min each) and incubated with IRDye 680CW fluorescent secondary antibody for 1 h at room temperature. After three washes in TBS-T again, the band intensity was visualized and quantified by the Odyssey Infrared Imaging System (Li-Cor Biosciences).

### Glutathione S-transferase pulldown assay

Glutathione S-transferase (GST) fusion proteins of EEA1 RBD-N (aa1-209), EEA1 RBD-C (aa1277–1411), Rabenosyn-5 RBD-N (aa1-40), and Rabenosyn-5 RBD-C (aa728–784) were expressed in *E. coli* BL21 *via* pGEX-4T-2 vector and affinity-purified by glutathione–Sepharose beads (GE Healthcare) (40). The pBI expression constructs of Rab5, Rab22, Rab31, and chimeras were transfected into indicated cell cultures, incubated for 24 h, and then lysed in lysis buffer containing 20 mM Hepes (pH 7.4), 100 mM NaCl, 5 mM MgCl_2_, 0.1% NP40, 10% glycerol, 1 mM dithiothreitol (DTT), and protease inhibitor cocktail (Sigma). The cell lysates were clarified by centrifugation at 10,000*g* for 2 min at 4 °C, and the supernatants were incubated with each GST–effector fusion protein on glutathione–Sepharose beads for 1 h at 4 °C (40). Beads were then washed 3 times with PBS-T (1% Triton X-100) and resuspended in SDS sample buffer, boiled for 3 min, and subjected to SDS-PAGE and for immunoblot analysis with anti-Myc or anti-Rab antibody as indicated.

### Confocal fluorescence microscopy

Cell monolayers were cultured on 10-cm coverslips in 24-well plates and transfected with pBI constructs expressing the indicated Rabs and chimeras, as described earlier. At 24 h post transfection, the cells on coverslips were fixed by 4% paraformaldehyde (w/v in PBS) for 15 min and permeabilized with 0.1% Triton X-100 (w/v in PBS) for 1 min at room temperature. Then, the cells were stained with antibodies for Rab22 (Abcam), Rab31 (Abcam), Rab5 (Calico Biolabs), EEA1, and GM130 (BD Biosciences), as indicated. After overnight incubation, the coverslips were stained with Alexa Fluor secondary antibodies 488 and 568 (Invitrogen) for 1 h at 37 °C, followed by DAPI staining and three washes in PBS. The coverslips were then mounted on glass slides with Prolong Gold anti-fade reagent (Invitrogen) for 24 h before imaging with Leica SP8 laser-scanning confocal microscope. Vesicle size and analysis was performed by diameter measurement, using LAS AF Lite software, of the largest vesicle in 10 individual cells of each group from triplicate samples. These measurements were then statistically analyzed with one-way ANOVA test using Prism software. For co-localization quantification of two fluorescence signals, Pearson’s correlation coefficients were calculated using the confocal images with the built-in co-localization tool in the Improvision Volocity software (PerkinElmer).

### GTP hydrolysis assay

The assay was conducted as previously described, with slight modifications. Briefly, the GST fusion proteins of Rab5 and Rab22 (1 μM) were bound to glutathione–Sepharose 4B resin and incubated with 0.1 μM [α-^32^P]GTP (PerkinElmer) for 30 min at 25 °C in 50 μl of loading buffer (20 mM Tris/HCl [pH 8]/2 mM EDTA/1 mM DTT). Unbound [α-^32^P]GTP was then removed by washing the resin twice with the same buffer. The GTP hydrolysis reaction was initiated by resuspending the resin in reaction buffer (20 mM Tris/HCl [pH 8]/5 mM MgCl/1 mM DTT) and incubating at 37 °C. Samples were taken at the indicated times and immediately solubilized in elution buffer (0.2% SDS/5 mM EDTA/5 mM GDP/5 mM GTP) by heating at 65 °C for 2 min. The eluted GTP and GDP were separated by TLC on polyethyleneimine-cellulose sheets (J. T. Baker) with 0.75 M KH_2_PO_4_, pH 3.5, as the developing solvent. The radioactive GTP and GDP spots were detected by autoradiography and quantified by using a phosphorimager (Molecular Dynamics).

### Subcellular fractionation

BHK cell monolayers grown in 35-mm culture dishes were transfected with the pBI constructs expressing Rab22, Rab31, Rab22/31C30, or Rab31/22C30. At 24 h post transfection, the cells were rinsed with ice-cold PBS and homogenized in 250 μl of TE buffer (100 mM Tris-HCl, pH 7.4, 1 mM EDTA) by passing through a 1-ml syringe with 25G5/8 needle 10 times. Cell lysates were centrifuged at 800*g* for 5 min to remove nuclei and cell debris, and postnuclear supernatants were subjected to high-speed centrifugation at 100,000*g* for 5 min in a Beckman-Coulter MAX-XP ultracentrifuge to separate the membranes (pellet) from the cytosol (supernatant). The membrane pellet was resuspended in the same volume of TE buffer as the cytosol fraction, and SDS (from 10% stock) was added to both fractions for a final concentration of 1%. The Rab construct in each fraction (20 μl) was analyzed by SDS-PAGE and immunoblot assay.

## Data availability

The data supporting the findings in this study are available within the article and supplemental information. Additional data are available upon request.

## Supporting information

This article contains supporting information.

## Conflict of interest

The authors declare that they have no conflicts of interest with the contents of this article.

## Supporting information

S1-Banworth et al Revision

Supp Fig 2 Banworth et al
