# Supplementary figures and images for: A novel membrane targeting domain mediates the endosomal or Golgi localization specificity of small GTPases Rab22 and Rab31
Source: J Biol Chem. 2022 Jul 19;298(9):102281. doi: 10.1016/j.jbc.2022.102281 (PMC9403361; doi:10.1016/j.jbc.2022.102281)

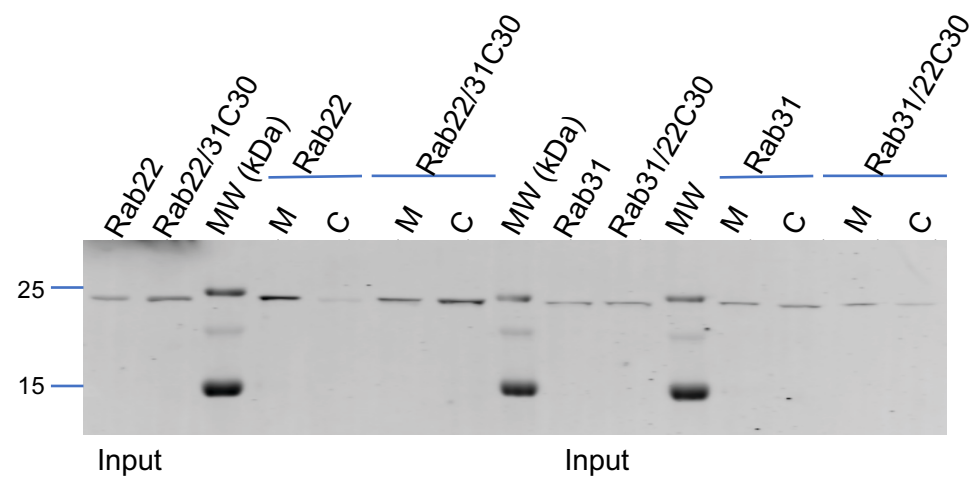

Supplemental Fig 1

Supplement: S1-Banworth et al Revision [file mmc1.pdf]

Rab5

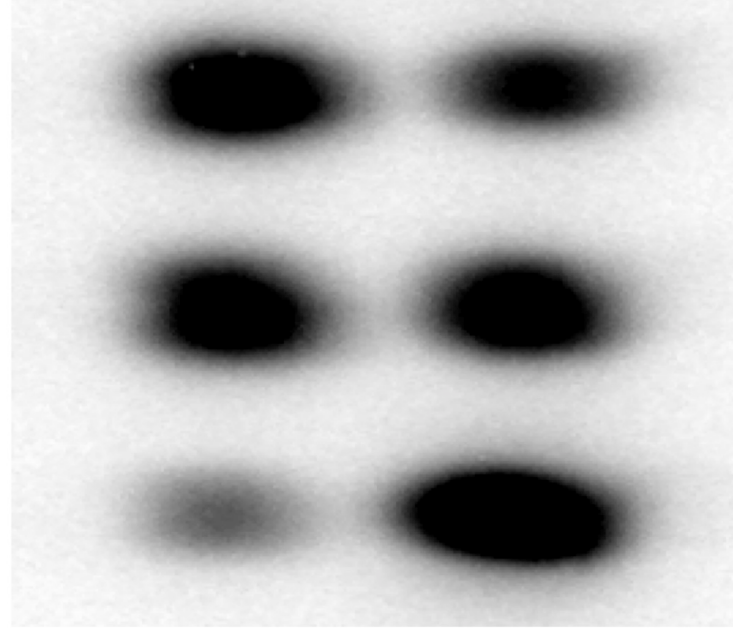

Time (Min)

0

10

20

Rab22

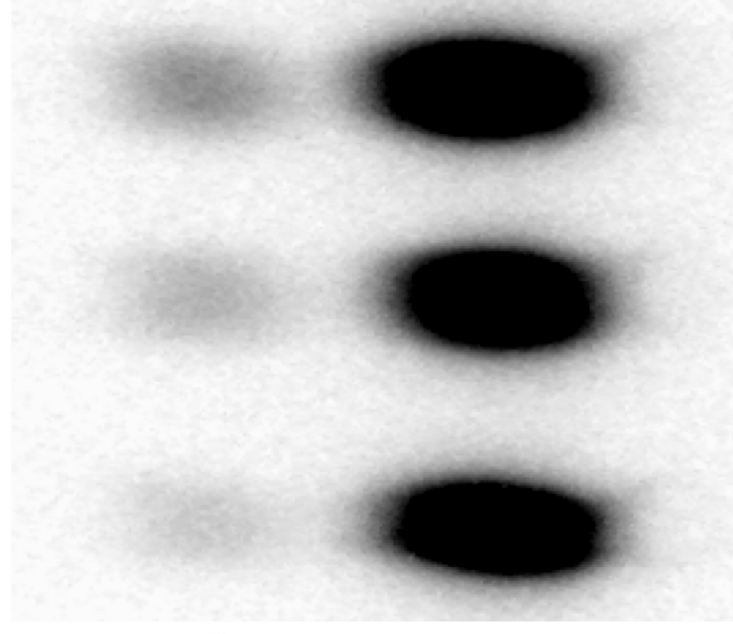

Time (Min)

0

10

20

Supplement: Supp Fig 2 Banworth et al [file mmc2.pdf]
